# Can training on ex-vivo models increase neurointerventionalists’ subjective self-confidence in the operating room?

**DOI:** 10.1371/journal.pone.0264180

**Published:** 2022-02-22

**Authors:** Nathalie Mathern, Johanna Sandmann, Thorsten Sichtermann, Hani Ridwan, Alexander Riabikin, Andrea Stockero, Omid Nikoubashman, Martin Wiesmann, German Stroke School Group

**Affiliations:** Department of Diagnostic and Interventional Neuroradiology, University Hospital RWTH Aachen, Aachen, Germany; Instituto Mexicano del Seguro Social (IMSS) HGZ 2, MEXICO

## Abstract

In a changing learning environment where young neurointerventionalists spend less time in the operating room, computer simulators have been established as a new training model. Our aim was the comparison of silicone models and computer simulators, and the evaluation of their influence on subjective self-confidence of operators. Pre- and postquestionnaires of 27 participants and 9 tutors were evaluated after the participation in a three-days interventional stroke course using silicone models and computer simulators. Training on computer simulators was considered as more realistic and important before patient contact than training on silicone models. Participants rated their own abilities as significantly better after participation in the course and felt significantly better prepared for patient care. Training on computer simulators can increase the subjective self-confidence of trainees. We suggest a stepwise training program, comprising both ex-vivo and the porcine in-vivo model, finished by conventional operating room teaching, to prepare neuroradiologists for optimal patient care when performing interventions.

## Introduction

Ischaemic stroke is one of the most frequent causes of death worldwide [1]. Endovascular stroke therapy has been established as the standard treatment option for emergent large vessel occlusion stroke. As a result, treatment has become more effective, but also more complex, necessitating more efficient training.

In the past, practical training of young neurointerventionalists took place in the operating room. However, the aim of increasing patient safety and increased complexity of neurointerventional procedures have led to the development of new training methods [2–4]. A major advantage of ex-vivo models is patient safety, as mistakes remain without clinical consequence [5–7]. By now, the most common training programs for neurointerventionalists consist of training on silicone and animal models. Computer simulators fill the gap between the opportunities, offered by these models. They are increasingly used in neurointerventional training because of their high degree of realism and the possibility to train various scenarios [8–11].

A major aim of such training programs is not only to train the actual procedure and to reduce complication rates, but also to increase the self-confidence of the trainee. To date, there are no studies that address the impact of computer-based simulators on subjective self-assessment. In this study we assessed whether computer-based simulators or silicone models improve subjective self-confidence of neurointerventionalists.

For this purpose, we prospectively evaluated an interventional stroke course, which offered hands-on training on silicone models and computer simulators, organized by the German Society of Neuroradiology (“German Stroke School”) held in Frankfurt, Germany, in October 2018. Using pre- and postquestionnaires, we asked participants whether their skills improved subjectively and whether they felt better prepared for patient care after joining this course. In a second part of the study we compared the two ex-vivo models by asking tutors and participants for their subjective assessment, hypothesizing that participants would benefit rather from computer simulators than from silicone models.

## Material and methods

### Endovascular training program

The three-days course took place in Frankfurt/Main in October 2018 and was aimed at young neurointerventionalists with first experience in intervention and angiography. Thirty-five national and international participants with different levels of experience in neuroradiology joined this course and were supervised by sixteen advanced tutors. The supervising tutors had several years of experience in interventional neuroradiology as well as in the use of computer simulators and silicone models.

The course consisted of a comprehensive lecture program and an intensive hands-on training on computer simulators and silicone models. To reduce the number of participants per group, the attendees were split up in two groups prior to participation. On the first day, they received a theory lesson and a short introduction in the use of silicone models and computer simulators. On the second and third day, four sessions per day were held: two practical and two theoretical sessions on various aspects of neurointerventional stroke therapy. During the practical parts, two attendees each trained on the silicone models and computer simulators under the guidance of one tutor. The practical training involved lessons about the access to supraaortal vessels, stent-retriever extraction, and carotid-stenting. Each session lasted a maximum of two hours with breaks in between.

### Questionnaires

Participants and tutors received two questionnaires to be filled out: one before and one after the course. Both questionnaires included questions in English language.

In the *prequestionnaire*, the respondents were asked how many years of experience they had, the number of thrombectomies they had assisted with, and the number of thrombectomies they had performed on their own. Furthermore, they were asked about their previous experience with silicone models and computer simulators and their opinion about the usage of these ex-vivo models in neurointerventional training. The respondents were also asked how realistic they expected the training on silicone models and computer simulators to be, how well they felt prepared for patient care before joining the course, and how they assessed their own abilities.

The *postquestionnaire* contained analogous questions to the prequestionnaire: The participants and tutors were asked about how realistic the training on silicone models and computer simulators was, and which model they would have liked to spend more time with. As in the prequestionnaire, the respondents were asked to assess their own abilities and whether they felt well prepared for patient care after participation in the course. They were also asked if they felt it to be important to train on ex-vivo models before performing thrombectomies in humans and which model they felt to be the most suitable one for such hands-on training. In addition, there was a question about whether their expectations about joining the course had been fulfilled.

### Data and statistical analysis

We used Chi-square, Fishers exact, and Wilcoxon test for the statistical analysis. P-values of an α level ≤.05 were considered as statistically significant. The statistical analysis was calculated in SPSS Statistics 25 software (IBM, Armonk, New York, USA).

For the statistical evaluation of the questionnaires, we evaluated the answers of the total group, the participants, and the tutors.

In a first part of the evaluation, we assessed whether the subjective self-confidence of the participants improved after joining the course. The group of participants was divided into participants with previous experience with computer simulator training (Group A) and those without previous experience with this kind of training (Group B). We determined, if there was a significant difference between the groups, and within a group before and after participation in the course.

For a comparison of both models, we investigated if there was a significant difference between the group of all participants and the tutors, in the second part of the evaluation. For questions asked before and after participation in the course and the comparison between both ex-vivo models, we also evaluated the differences within a group.

### Ex-vivo models

#### Silicone models

For the practical training two silicone models were provided. The silicone models (Neuro Testing Model Plus, United Biologics, Santa Ana, California, USA) were filled with distilled water, with addition of shampoo to reduce friction of the devices on the silicone. A pulsatile pump was connected to imitate blood flow. The models were made of soft and transparent silicone, which enabled the participant to practice under direct visualization. A camera and a screen were used to enable indirect visualization. The silicone models include the aorta, the supraaortic branches, the circle of Willis, and the proximal segments of the anterior, middle and, posterior cerebral artery (A2, M2, P2). Training comprised catheterization of the aortic arch and the intracranial arteries as well as stent-retriever deployment.

#### Computer simulators

Twelve computer simulators (VIST G5, Mentice, Gothenburg, Sweden) were used. The vascular intervention system trainer (VIST) is a state of the art computer-based system that uses a geometric vessel representation which allows a high degree of realism for the user. Actual endovascular devices used in clinical practice, such as standard microcatheters, guidewires, and stent-retrievers can be applied in these computer simulators. In this course the clinical software module “Acute Ischemic Stroke Intervention” was used (Mentice, Gothenburg, Sweden). Scenarios comprised thrombectomies in different branches using stent-retrievers and balloon-guide catheters.

This study was part of our continuous quality control procedures and as such approved by the Ethics Committee of the University Hospital RWTH Aachen. Need for consent of participants was waived since the questionnaires did not contain any personal information and were thus fully anonymous.

## Results

We received a total of 39 questionnaires. Thirty-six were completely filled in, of which nine (25%) were completed by tutors and 27 (75%) by participants.

### Evaluation of the course

For the first part of the evaluation, the group of participants was split into those with previous experience with computer simulator training (Group A: 13 participants, 48%) and those without previous experience with this kind of training (Group B: 14 participants, 52%). Tables 1 and 2 provide the answers of all participants and the comparison between and within group A and B.

**Table 1:** Illustration of the statistical evaluation of the received questionnaires.

Participant indicated that	Total	Group A	Group B	p-value
He/she had experience with silicone models before the course	15/27 (56%)	10/13 (77%)	5/14 (36%)	.031
His/her abilities were good before the course	11/27 (41%)	6/13 (46%)	5/14 (36%)	.581
His/her abilities were good after the course	15/26 (58%)	9/13 (69%)	6/14 (43%)	.334
He/she felt well prepared for patient care before the course	6/27 (22%)	4/13 (31%)	2/14 (14%)	.385
He/she felt well prepared for patient care after the course	23/26 (89%)	11/13 (85%)	12/14 (86%)	1.000
His/her abilities in the computer simulator match/are better than his/her abilities in real life	22/27 (81%)	10/13 (77%)	12/14 (86%)	.648

The agreement to the statement in the left column of the total group, group A, and group B is given in relative and absolute numbers. The p-value in the right column is related to the difference between both groups.

**Table 2:** Illustration of differences within a group.

Participant indicated that	p-value Group A	p-value Group B
His/her own abilities were good **before/after** the course	.083	.257
He/she felt well prepared for patientcare **before/after** the course	.008	.001

Comparison of the questions asked before and after participation in the course. The p-value is related to the difference within a group.

**Fig 1:**
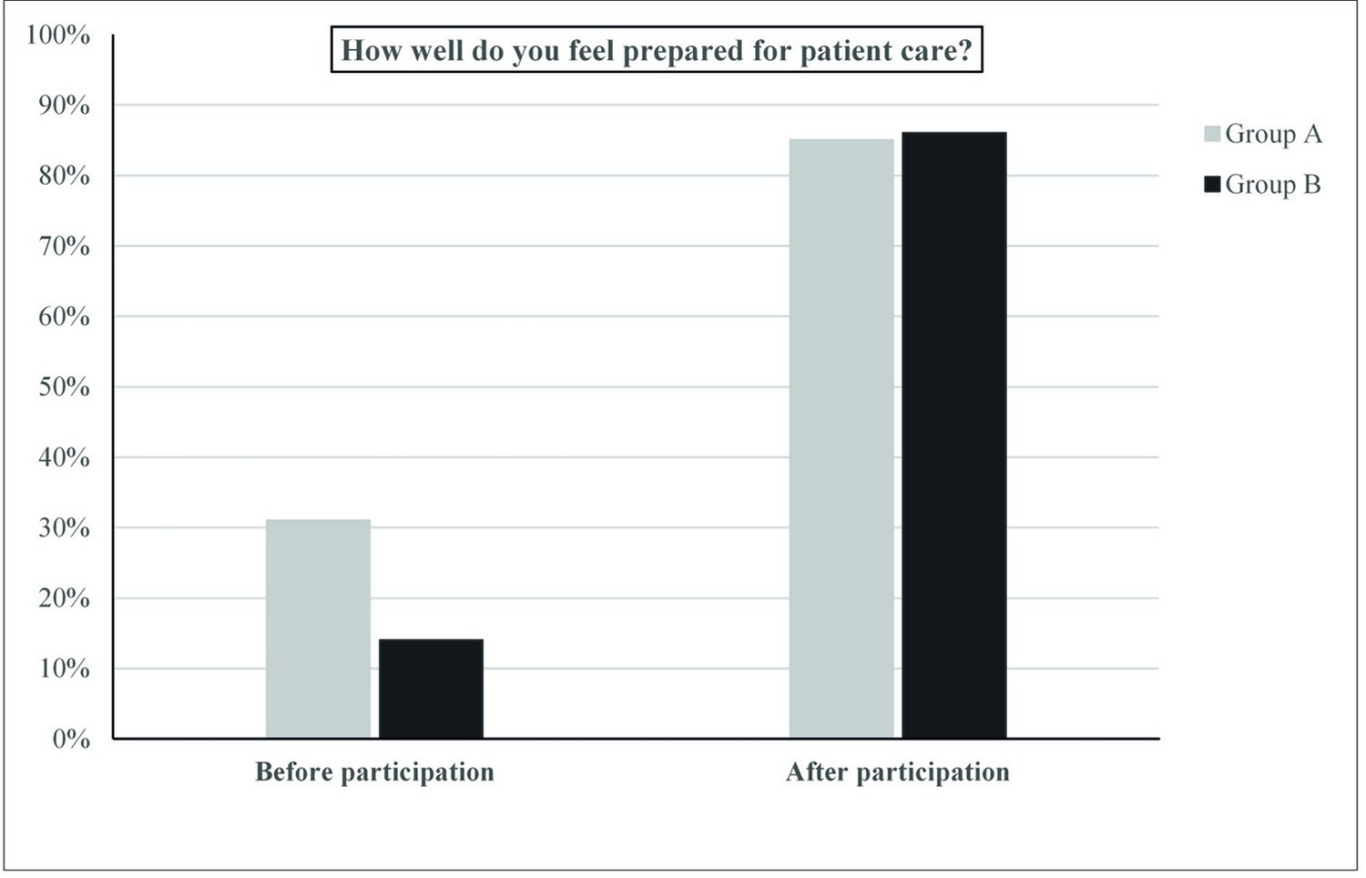
Graphical representation of the question how well prepared the participants felt for patient care before and after taking part in the course. Before joining the course, 31% of participants with previous experience with computer simulators (group A) and 14% of participants without previous experience with computer simulators (group B) indicated that they felt well prepared for patient care. Afterwards, 85% of group A, and 86% of group B agreed with this statement. There was a significant difference before and after participation in this course within both groups (Group A: p = 0.008, group B: p = 0.001).

We found a significant difference between the groups (p = 0.031) when the participants were asked about their previous experience with training on silicone models (Group A: 77%, Group B: 36%). Before joining the course, 31% of group A and 14% of group B indicated that they felt well prepared for patient care. Afterwards, 85% of group A, and 86% of group B agreed with this statement. There was a significant difference before and after participation in this course within both groups (Group A: p = 0.008, group B: p = 0.001; Fig 1).

### Computer simulator versus silicone model

Tutors and participants were asked to evaluate computer simulators and silicone models (Tables 3 and 4): Silicone models were considered to be realistic by 91% and 86% of all respondents before and after joining the course, respectively.

**Table 3:** Illustration of the statistical evaluation of the received questionnaires.

Participant/tutor indicated that	Total	Participant	Tutor	p-value
It is realistic to train on silicone models (statement **before** the course)	32/35 91%	24/27 89%	8/9 89%	1.000
It was realistic to train on silicone models (statement **after** the course)	30/35 86%	22/27 82%	8/9 89%	1.000
It is realistic to train on computer simulators (statement **before** the course)	31/35 89%	22/26 84%	9/9 100%	.665
It was realistic to train on computer simulators (statement **after** the course)	34/35 97%	27/27 100%	8/9 89%	.250
It was important to train on **silicone models** before doing thrombectomies in humans	24/31 77%	17/24 71%	7/7 100%	.161
It was important to train on **computer simulators** before doing thrombectomies in humans	33/33 100%	26/26 100%	7/7 100%	n/a
He/she wanted to spend more time on the **silicone model**	12/34 35%	11/26 42%	1/8 13%	.210
He/she wanted to spend more time on the **computer simulator**	28/35 80%	24/28 86%	4/8 50%	.033

The agreement to the statement in the left column of the total group, the participant, and the tutor group is given in relative and absolute numbers. The p-value in the right column is related to the difference between the participant and tutor group.

**Table 4:** Illustration of the statistical differences within a group.

Participant/tutor indicated that	p-value participant	p-value tutor
It is realistic to train on **silicone models/computer simulators** (statement **before** the course)	.317	.317
It was realistic to train on **silicone models/computer simulators** (statement **after** the course)	.480	.180
It was important to train on **silicone models/computer simulators** before doing thrombectomies in humans	.008	1.000
He/she wanted to spend more time on **silicone model/computer simulator**	.001	.083

The p-value is related to the difference within a group for the respective comparison listed in the left column.

Before attending the course, training on computer simulators was assessed to be realistic by 84% of the participant and 100% of the tutor group. Afterwards, all participants (100%) and almost all tutors (89%) agreed with this statement. Training on silicone models was considered to be important by 77% (71% of the participant group, 100% of the tutor group, p = 0.161) and training on computer simulators by 100% of both groups. We found a significant difference within the group of participants (p = 0.008), when we compared how important they assessed the training on silicone models or computer simulators before doing thrombectomies in humans.

After the course, 35% of all respondents indicated that they wanted to spend more time on the silicone model (43% of the participants, 13% of the tutors). By contrast, 80% of all respondents, 86% of the participant group, and 50% of the tutor group agreed to the statement that they would have liked to spend more time on the computer simulator. When comparing the two models, a significant difference was found within the participant group (p = 0.001).

All data that support the findings of this study are provided in full detail in the supporting information files.s

## Discussion

Our results show that training on silicone models and computer simulators is considered to be useful before patient care.

Tutors and participants rated the training on computer simulators as more important and realistic than training on silicone models. Silicone models offer a simplified training setting compared to the computer simulator, because direct visualization is possible. This is in line with previous studies, which have shown that silicone models are particularly helpful for the training of basic skills [12].

Simulators provide a broad spectrum of scenarios and uniform standards, which makes them effective ex-vivo models in endovascular training for novice and advanced neurointerventionalists. It is a predominant advantage of simulators, that surgical skills can be acquired in a safe environment [13,14]. Techniques can be practiced and repeated before they are applied to the patient. Unlike the silicone model, simulation-based training allows to carry out the most common procedures, such as guidewire and catheter navigation, but also more complex scenarios like carotid artery stenting [10,15]. Tutors rated the computer simulator training as more important and realistic than the training on silicone models. For experienced neurointerventionalists who have already basic knowledge, computer simulation offers the advantage of training complication management, expanding own skills, and learning new procedures. In addition, the use of the latest generation of simulators enables the objective assessment of key competencies and the evaluation of one’s own improvement over time [16].

Our results show that training on silicone models and computer simulators improves self-confidence of aspiring neurointerventionalists. Previous training with ex-vivo and porcine in-vivo models can help the trainee to feel more comfortable in the operation room, which is often perceived as more stressful than the educational setting. Pre-acquired basics in all available models can prepare the trainee for optimal patient care. Stolarek et al. claimed that intensive simulation-based training can be as effective as clinical experiences [17]. Other studies state that ex-vivo models will not be able to replace the operating room as a training area for residents [4,17]. In our opinion, the ideal training consists of a combination of ex-vivo and in-vivo models and the conventional operating room training. A training for neurointerventional residents should include a sort of pretraining, ensuring that the trainee has a theoretical understanding of the skills, the devices, and the correct methods of the performance [2]. After the theoretical part, basic skills and new techniques should be trained on silicone models. Computer simulation training allows first processing complete procedures. Liebig et al. even demand, that trainees must reach a certain level of proficiency on the computer simulator, before treating a real patient [18]. Porcine in-vivo models could therefore be the last step, completed by the final training in the operating room on real patients. For ethical reasons, porcine in-vivo models should be used after solid basic skills have been acquired in both ex-vivo models. All these training models should not be seen as a replacement for traditional residents training, but as an essential addition [18].

## Limitations

The ideal measure for the effectiveness of training is not self-confidence but improved clinical skills and ultimately improved clinical outcome. As participants of our course came from different clinics and countries with different practices and infrastructure, it was impossible to assess whether our training had an impact on clinical outcome of subsequent patients. An alternative to measuring the real-life performance would be testing the performance with varying simulation scenarios. However, this involves the risk of testing specifically trained simulation-skills instead of real-life skills. In summary, the transferability of simulation-learnt skills to clinical practice is a major limitation of simulation-based training, which has not been addressed sufficiently in the literature [19,20]. This is why we focused our analysis on self-assessment as a subjective, but reliable factor. However, even though an increase in self-confidence is generally perceived as an advantage in clinical practice, we see a risk in the discrepancy between the actual ability and subjective self-assessment. Recurring scenarios and repetitions of the same exercises may lead to increased self-confidence and a false sense of security.

## Conclusion

We come to the conclusion that a variety of simulators should be used. Importantly, both tutors and participants rated the training on computer simulators as more important and realistic than training on silicone models. Therefore, a fixed part in the training curriculum of neurointerventionalists should be computer simulators as they provide many benefits. Residents today have to deal with increased patient safety standards and the effect of time constrains in their education. In this changing learning environment, simulation based learning could fix the gap between theory and practice [17].

## Supporting information

S1 File (XLSX)
